# General Anesthesia Improves Efficiency of High-Power Short-Duration Catheter Ablation for Atrial Fibrillation: Comparison with Mild Conscious Sedation

**DOI:** 10.3390/jpm14080865

**Published:** 2024-08-16

**Authors:** Ioan-Alexandru Minciună, Raluca Tomoaia, Mihai Suceveanu, Gabriel Cismaru, Mihai Puiu, Radu Roșu, Gelu Simu, Diana Andrada Irimie, Florina Frîngu, Bogdan Caloian, Marius Andronache, Dumitru Zdrenghea, Dana Pop

**Affiliations:** 1 5th Department of Internal Medicine, Faculty of Medicine, Iuliu Hațieganu University of Medicine and Pharmacy, 400347 Cluj-Napoca, Romania; minciuna.ioan.alexandru@elearn.umfcluj.ro (I.-A.M.); rosu.radu1053@elearn.umfcluj.ro (R.R.); simugelu@yahoo.com (G.S.); gurzau.diana@elearn.umfcluj.ro (D.A.I.); florina.fringu@elearn.umfcluj.ro (F.F.); bogdan912@elearn.umfcluj.ro (B.C.); dzdrenghea@elearn.umfcluj.ro (D.Z.); dana.pop@umfcluj.ro (D.P.); 2 Cardiology Department, Rehabilitation Hospital, 400066 Cluj-Napoca, Romania; suceveanu.paul.mihai@elearn.umfcluj.ro (M.S.); puiu.mihai@yahoo.com (M.P.); 3 Alleray-Labrouste Cardiology Clinics, 75015 Paris, France; marius.andronache@elearn.umfcluj.ro

**Keywords:** atrial fibrillation, catheter ablation, high-power short duration, general anesthesia, mild conscious sedation

## Abstract

Background: Atrial fibrillation (AF) is the most common cardiac arrhythmia globally. High-power, short-duration radiofrequency (RF) catheter ablation (CA) for AF has recently emerged, reducing ablation times and enhancing patient tolerability with comparable efficacy and safety. While the benefits of general anesthesia (GA) for standard-power, standard-duration CA are well-established, data comparing GA to mild conscious sedation (MCS) for high-power, short-duration CA are limited. Methods: We included patients undergoing high-power, short-duration CA for AF under GA (group 1) or MCS (group 2). Procedural characteristics, success rates, and mid-term outcomes were compared. Results: In total, 131 patients, 47 in the GA group and 84 in the MCS group, were included. CA was performed for paroxysmal AF in 34 patients in group 1 (72.3%) and 68 patients in group 2 (80.9%). We found lower a mean total procedure time (100 [90–120] vs. 160 [130–180] min, *p* < 0.0001), lower radiation exposure (932.5 [625–1716] vs. 2445 [1228–4791] μGy, *p* < 0.0001 and 4.5 [3–7.1] 7.3 [4.2–13.5] min, *p* = 0.0003) and fewer RF applications (71 [54.8–83.8] vs. 103 [88.5–120.5], *p* < 0.0001) in the GA group. No major complications occurred. The 6-month AF recurrence rate was comparable between the groups (21.2% vs. 33.3%, *p* = 0.15). Conclusion: In patients undergoing high-power, short-duration RFCA for AF, the use of GA is associated with better procedural efficiency while simultaneously associated with an early recurrence rate comparable to MCS.

## 1. Introduction

Atrial fibrillation (AF) represents the most common cardiac arrhythmia in adults worldwide and is associated with a significant increase in morbidity, mortality, and socioeconomic burden. Over the past few decades, radiofrequency (RF) catheter ablation (CA) has become a widely adopted treatment option for symptomatic AF [1,2,3,4]. Continuous advancements in ablation techniques are being developed to enhance procedural efficiency and optimize patient outcomes. Recently, the high-power short-duration (HPSD) approach has emerged as an alternative to standard-power standard-duration settings, showing reduced ablation times and increased patient tolerability, with similar outcomes in terms of safety and efficacy [5,6].

CA for AF is usually performed under general anesthesia (GA) or mild conscious sedation (MCS), depending on the operator’s preference, the patient’s characteristics, and the center’s facilities [7,8]. Several studies have compared the efficacy and safety of AF catheter ablation under either GA or MCS [4,7,8,9]. The 2024 multi-society expert consensus statement on the catheter and surgical ablation of AF highlights that GA is frequently preferred over MCS. The advantages of GA include minimized patient movement, improved contact force and catheter stability, lower radiation exposure, and greater first-pass isolation. These factors are associated with significantly improved ablation efficacy, while the safety profile remains comparable to MCS. Although the advantages of GA are well established for standard-power standard-duration AF ablation, there are currently a few reports describing the anesthesia protocol for the HPSD approach [10,11]. To our knowledge, no data exist in the literature comparing procedural outcomes using GA versus MCS for HPSD CA for AF.

The aim of this study was to determine the impact of GA compared to MCS on the procedural efficiency, efficacy, and safety of HPSD RFCA for AF.

## 2. Method

### 2.1. Patient Population

A series of 131 patients undergoing RFCA for paroxysmal or persistent AF were enrolled between 2017 and 2021 at two different institutions. This cohort was extracted from a total group of 321 patients who received AF ablation during the study period. We excluded 123 patients who underwent AF ablation using cryoenergy and 67 patients in whom RFCA was performed using standard-power settings. The cohort consisted of 87 males (66.4%) and 44 females (33.6%). The mean age of the study group was 59.2 ± 10.4 years (61.3 ± 9.5 for females and 58.1 ± 10.8 for males). Paroxysmal/persistent AF was defined according to the 2020 European Society of Cardiology Clinical Practice Guidelines for the Management of Atrial Fibrillation. All patients were effectively anticoagulated. Transesophageal echocardiography and computed tomography angiography were performed in all patients prior to CA in order to exclude left atrial thrombus and assess pulmonary veins and left atrium anatomy. The study was approved by the local ethics committee. All included patients signed written informed consent forms before the procedure.

### 2.2. Ablation Procedure

Ablations were performed by 4 experienced electrophysiologists using an irrigated-tip, contact-force sensing catheter (Thermocool SmartTouch SF, Biosense Webster, Diamond Bar, CA, USA) and a multielectrode mapping catheter (PentaRay NAV, Biosense Webster, Diamond Bar, CA, USA). A transseptal puncture was guided using fluoroscopies for all patients. After transseptal punctures, an intravenous bolus of 5 to 10.000 units of heparin was administered, followed by continuous infusion depending on the weight of the patient to achieve activated coagulation times between 300 and 350 ms. Electroanatomical mapping was performed using the Carto system (Biosense Webster, Diamond Bar, CA, USA). The power settings used were 50 W with an ablation index of 450 for the anterior wall and 320 for the posterior wall. Esophageal temperature was not directly monitored. The primary endpoint of the procedures was the elimination of all pulmonary vein potentials along the antra (entry and exit block). Procedural safety was evaluated by intra- and post-procedural complications. Procedural efficiency was evaluated by total procedural time, the number of radiofrequency applications, fluoroscopy time, and dose. Procedure start and finish times were defined as the time from femoral puncture to catheter removal from the body.

### 2.3. Anesthesia Protocol

The same four experienced electrophysiologists performed the ablations under GA in one of the hospitals (group 1, 47 patients) and under MCS in the other institution (group 2, 84 patients). Patients with contraindications to either GA or MCS were excluded from the study. In group 1, anesthesia induction involved administering 2 mg/kg propofol and 1 to 2 micrograms/kg fentanyl, followed by a non-depolarizing muscle relaxant, typically rocuronium 0.6 to 1 mg/kg, followed by endotracheal intubation. Anesthesia was maintained with inhaled anesthetics delivered via an endotracheal tube and intermittent positive-pressure ventilation. In group 2, MCS was achieved with intravenous morphine and midazolam administered by the nursing staff without the participation of an anesthesiologist.

### 2.4. Follow-Up

Patients were discharged after one to three days of observation. Follow-up evaluations were conducted at 3- and 6-months post-procedure and included a cardiology assessment, a 12-lead electrocardiogram, and 24 h Holter monitoring. Antiarrhythmic drugs were continued for 3 months after the procedure, representing the blanking period. AF recurrence was defined as any episode of AF/atrial tachycardia lasting more than 30 s after the blanking period.

### 2.5. Statistical Analysis

The baseline characteristics of the patients were reported as the mean ± standard deviation (SD) for quantitative variables that followed a normal distribution, and the median [interquartile range (IQR)] for quantitative variables that followed abnormal distribution or frequencies (percentages) for categorical variables. Normality was assessed using the Kolmogorov–Smirnov test for all subjects and in separate groups. Patients were categorized based on the use of GA or MCS. Continuous variables were analyzed using *t*-tests or Mann–Whitney U tests, depending on their type and distribution while categorical data were compared using the Chi-square test. Clinically representative parameters were presented as box-and-whisker plots. The prognostic value of each clinical, echocardiographic, and procedural variable in predicting the development of AF recurrence was determined using univariate and multivariate logistic regression analysis. To exclude potential confounders, multivariate regression analysis was performed for all patients, subsequently following an analysis based on the AF type. The parameters were only regarded as significant in the multivariate analysis if *p* < 0.05. A post hoc power analysis for AF recurrence (Chi-squared test) was performed based on the proportion of AF recurrence in each group, using an alpha level of 0.2 and a power of 80%.

Statistical analysis was conducted using the MedCalc Statistical Software version 22.016 (MedCalc Software Ltd., Ostend, Belgium; http://www.medcalc.org (accessed on 30 of October 2022). A *p*-value of <0.05 was considered significant.

## 3. Results

The study population consisted of 131 patients with either paroxysmal or persistent AF who underwent HPSD CA for AF using either GA (group 1, n = 47) or MCS (group 2, n = 84). CA was performed for paroxysmal AF in 34 patients in group 1 (72.3%) and 68 patients in group 2 (80.9%) and for persistent AF in the rest of the patients. There were no significant differences between the two groups regarding baseline clinical characteristics, antiarrhythmic medication use, or left atrial diameter as evaluated by echocardiography. Patient clinical and procedural characteristics are summarized in Table 1.

The acute procedural endpoint was achieved in all patients (100%). Additional non-pulmonary vein triggers were targeted in 11 patients in group 1 (23.4%) and 18 patients in group 2 (20.68%), all with persistent AF. Cavotricuspid isthmus ablation was performed in 6 patients in group 1 (12.76%) and in 3 patients in group 2 (3.57%).

**Figure 1:**
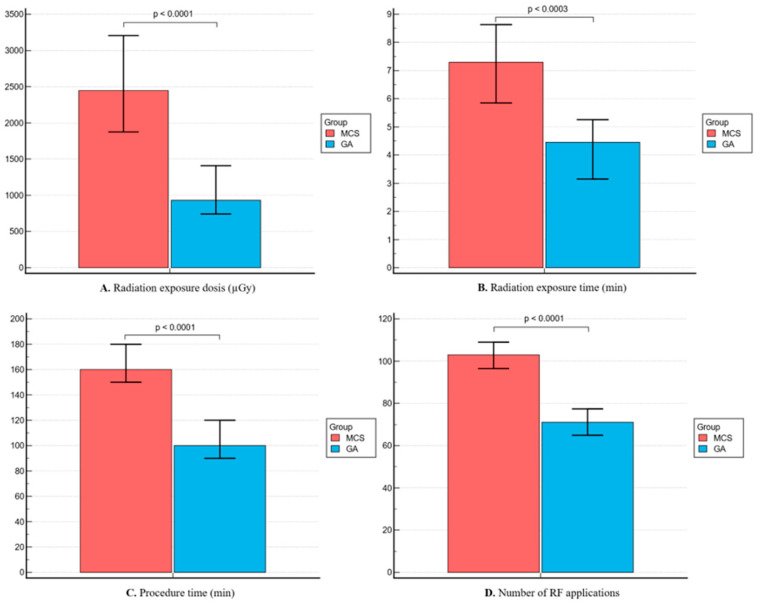
Comparison of procedural efficiency parameters between the two groups. (**A**) Radiation exposure—dosis (mGy); (**B**) radiation exposure—time (min); (**C**) total procedural time (min); and (**D**) number of radiofrequency (RF) applications.

**Figure 2:**
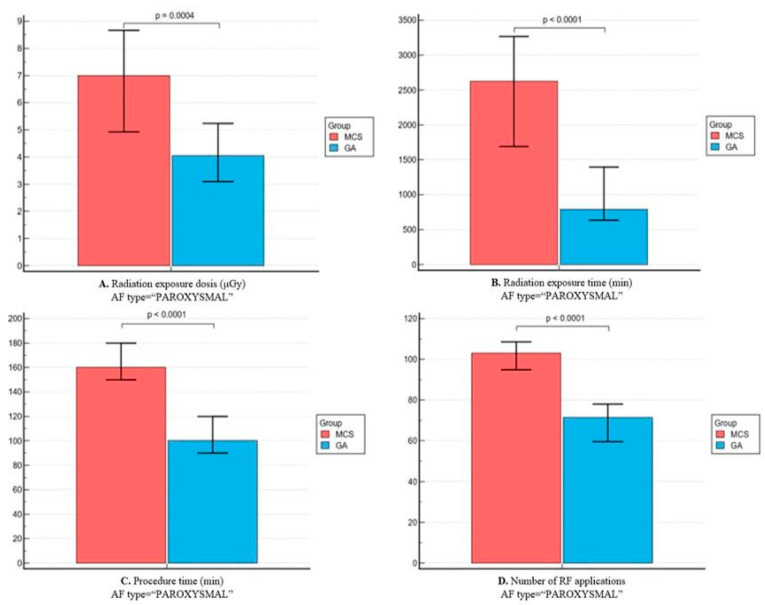
Comparison of procedural efficiency parameters between the two groups for paroxysmal AF patients. (**A**) Radiation exposure—dosis (mGy); (**B**) radiation exposure—time (min); (**C**) total procedural time (min); and (**D**) number of radiofrequency (RF) applications.

**Figure 3:**
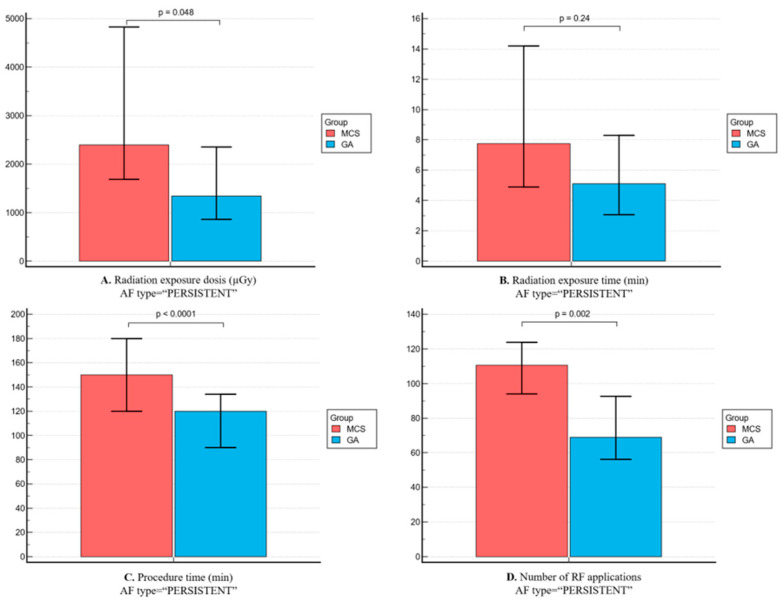
Comparison of procedural efficiency parameters between the two groups for persistent AF patients. (**A**) Radiation exposure—dosis (mGy); (**B**) radiation exposure—time (min); (**C**) total procedural time (min); and (**D**) number of radiofrequency (RF) applications.

As shown in Figure 1, group 1 had a lower mean total procedure time (100 [90–120] vs. 160 [130–180] min, *p* < 0.0001), lower radiation exposure (932.5 [625–1716] vs. 2445 [1228–4791] μGy, *p* < 0.0001 and 4.5 [3–7.1] 7.3 [4.2–13.5] min, *p* = 0.0003) and lower number of RF applications (71 [54.8–83.8] vs. 103 [88.5–120.5], *p* < 0.0001). Correspondingly, among patients with paroxysmal AF, patients from group 1 showed lower radiation exposure and shorter procedural and fewer RF applications, whereas in patients with persistent AF, radiation exposure times were similar between the two groups (Figure 2 and Figure 3).

**Figure 4:**
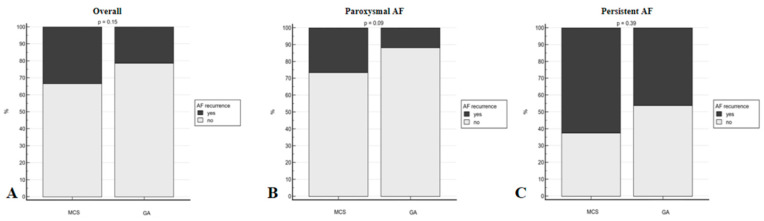
Comparison of AF recurrence at 6-month follow-up between the two groups. (**A**) Overall; (**B**) paroxysmal AF; and (**C**) persistent AF.

All patients were followed-up using the strategy described in the Section 2. AF recurrence at the 6-month follow-up was lower in group 1 compared to group 2 (21.2% vs. 33.3%); however, this did not reach statistical significance (*p* = 0.145). Similar results were observed for both paroxysmal and persistent AF patients (Figure 4).

There were no complications related to the general anesthesia or the anesthetic agents used in this patient population. There was one moderate pericardial effusion in the MCS group, which resolved with pharmacological treatment. No other major complications, including pericardial effusion/tamponade requiring pericardiocentesis or surgery, atrioesophageal fistula, or stroke, were observed in either group.

In the univariate logistic regression analysis, we found that only left atrial diameter (OR 1.18, 95%CU 1.08 to 1.28, *p* = 0.0003), the number of RF applications (OR 1.01, 95%CI 1.0009 to 1.03, *p* = 0.03) and persistent AF (OR 4.447, 95%CI 1.97 to 10.69, *p* = 0.0007) were associated with early AF recurrence, while the type of anesthesia was not (Table 2). Among the clinical characteristics, only the presence of more than mild mitral regurgitation was associated with AF recurrence.

By considering the type of anesthesia used with the presence of more than mild mitral regurgitation, the left atrial diameter, the number of RF applications, and the type of AF, a multivariate logistic regression was applied to assess early AF recurrence (Table 3). We found that only persistent AF (OR 3.33, 95%CI 1.19 to 9.32, *p* = 0.02) was independently associated with AF recurrence. Considering the well-established higher risk of recurrence associated with persistent AF, we tested the same variables according to the type of AF. We found that only the left atrial diameter was independently associated with AF recurrence in patients with paroxysmal AF, whereas none of these parameters were present in persistent AF patients. It is noteworthy that the type of anesthesia used was not correlated with a higher recurrence of AF in either group.

The post hoc power analysis for AF recurrence demonstrated that a total sample of 271 patients, including 98 in the GA group and 173 in the MCS group, would be required to achieve a power of 80%.

## 4. Discussion

This is the first study comparing GA to MCS for AF CA using a HPSD approach. The main findings of the present study are as follows: (1) GA improves procedural efficiency by lowering the total procedure time and fluoroscopy time, and dose; (2) the total number of RF applications was lower when GA was used, showing that catheter stability and lesion formation improves with GA; (3) there were no differences in acute success rates between GA and MCS; (4) patients on whom GA was used showed a tendency toward lower risk of recurrence during follow-up compared to MCS; (5) the prevalence of perioperative complications was similar between GA and MCS, with no major complications using either approach; and (6) no anesthesia-related complications were observed using either GA or MCS.

RFCA has been shown to be safe and effective for AF treatment, but the procedures tend to be long and complex and can cause patient discomfort [1,9]. During the procedure, variations in respiration and patient movement caused by pain can affect catheter stability and the quality of signals and lesion formation, which can impact both procedure efficacy and safety [9,12,13]. Conscious sedation and GA are commonly used for electrophysiological procedures in order to improve procedural outcomes and increase patient comfort [14]. Most of the time, choosing between the two strategies depends mainly on the operator’s preference, the patient’s characteristics, and the center’s resources [7,8,14]. Compared to GA, conscious sedation does not require access to a dedicated cardiac anesthetist, which results in less resource consumption. Also, there are no intubation-related injuries and anaphylaxis-related complications. On the other hand, GA offers the advantages of pain elimination during the procedure and assisted ventilation, allowing better mapping and increased catheter stability [12,13,15]. In this study, the choice of GA versus MCS was completely dependent on the two different institution’s facilities and resources.

Over time, multiple studies have shown the advantages of GA compared to MCS for AF RFCA using standard-power standard-duration settings [7,8,9,12,13,16]. The 2024 multi-society expert consensus statement on the catheter and surgical ablation of AF summarizes that GA is usually preferred over MCS, given the need to minimize patient movement to improve catheter and mapping system stability. Despite this, there are no clear guidelines or recommendations for the anesthesia strategy during RFCA for AF [3]. Also, as techniques for CA continue to evolve, anesthesia requirements are continuously changing, making it even more challenging to establish clear recommendations.

Since the introduction of the HPSD approach for AF ablation, there have been a few reports describing the anesthesia protocol during the procedure. Most centers prefer GA for HPSD CA, considering the increased patient discomfort when using this approach [10,11,17]. In a study of patients undergoing very HPSD RFCA (90 W) for AF, Chu and colleagues showed that very HPSD pulmonary vein isolation can be performed under MCS without compromising patient experience [10]. Similar results were presented by Poggi et al. at the European Heart Rhythm Association Congress in 2023 [11]. However, to date, there are no data in the literature comparing GA to MCS for HPSD CA for AF. This is the first study that looks at the differences in terms of procedural efficiency, efficacy, and safety between the two different anesthesia strategies since the introduction of HPSD RFCA for AF.

The total procedure time and radiation exposure can vary between GA and MCS depending on several factors, including the anesthesia protocol and different definitions between studies [4,7,8,9,16,18]. In this study, GA was associated with shorter total procedure times and lower fluoroscopy exposure (time and dose) in patients undergoing HPSD RFCA for AF. These results are consistent with previous reports by Di Biase and colleagues using the standard-power standard-duration approach for paroxysmal AF RFCA [7,9]. Also, Yokokawa et al. found lower fluoroscopy exposure with GA compared to MCS but with longer total procedure duration for GA. However, this could be explained by the anesthesia protocol, which required the involvement of a dedicated anesthesiologist for the conscious sedation group as well. In fact, in their study, complete pulmonary vein isolation was achieved faster in the GA group, with the longer procedure times being related to longer anesthesia care [8,18]. Other studies that compared GA to MCS, including a meta-analysis and systematic review by Christensen Li and colleagues, found no significant differences in total procedure time and fluoroscopy time between these two strategies. Nevertheless, the authors also mention that this is most likely related to the longer anesthesia duration in the GA group and not to longer ablation times [4,7,16].

Several studies have reported data on the acute and mid- or long-term efficacy of GA and MCS during standard power duration RFCA for AF [4,7,9,18]. As mentioned previously, GA provides better respiration control and catheter stability, which may facilitate continuous and durable lesion creation [18]. In the present study, acute procedural success was achieved in all patients with the use of the HPSD approach (100%), regardless of the anesthesia strategy. The number of RF applications was significantly lower in the GA group compared to the MCS group. In a study by Chikata et al., the lower number of RF applications and gaps between points with the use of GA was associated with better contact force, translating into better catheter stability and lesion formation [7].

Previous studies on the relationship between anesthesia strategy and AF recurrence have yielded conflicting results. It has been reported that GA reduces AF recurrence and the prevalence of pulmonary vein reconnection when compared to MCS in patients with both paroxysmal and persistent AF [4,7,9,16]. In a study by Di Biase and colleagues, patients undergoing RFCA for paroxysmal AF who were randomized to GA were more likely to remain free of atrial arrhythmias and had fewer reconnected pulmonary veins during repeat ablation compared to MCS [9]. Also, Chikata et al. demonstrated that GA is associated with improved long-term outcomes in patients with paroxysmal AF but not with persistent AF [7]. Martin et al. confirmed these results for the RFCA of persistent AF, showing that GA rather than sedation is both clinically and economically more effective [16]. In contrast, Yokokawa et al. reported no significant differences in terms of AF recurrence between GA and MCS [18]. Also, in a study of patients undergoing cryoballoon CA for paroxysmal AF, Wasserlauf, and colleagues found that both GA and MCS groups had similar rates of freedom from AF [19]. In our study, GA showed a tendency to lower the 6-month recurrence rate compared to MCS, though this was statistically insignificant, which is similar to the results reported by Christien Li et al. [4].

In the past, GA had limited use due to its potential complications. Currently, thanks to advances in technology and the advent of new medications, GA is considered safe. In our study, the prevalence of perioperative complications was similar between the two groups. There was no atrioesophageal fistula or tamponade with either approach. Also, there were no complications related to anesthetic agents or anesthesia in the present study. Similar results are reported in the literature, demonstrating a reduced incidence of perioperative and anesthesia-related complications with both GA and MCS [4,7,9,16,18].

## 5. Study Limitations

The main limitation of this study is that it is a single-center, retrospective, non-randomized analysis. It was, therefore, not possible to control different operator techniques and decision-making based on individual patient characteristics. Second, the 6-month follow-up period was shorter compared to other studies. This is due to the high number of patients lost during 12- and 18-month follow-ups. Third, we did not evaluate esophageal tissue damage. A higher risk of esophageal damage has been reported to be associated with GA compared to MCS, likely due to the decrease in deglutition and reduction in the cooling effect from swallowing [4,7]. Although there were no clinical signs of esophageal damage or atrioesophageal fistula with either approach, we cannot exclude the possibility of some esophageal damage in any of the groups. Lastly, the relatively small sample size may account for the observed trend of lower AF recurrence in the GA group, although this did not reach statistical significance. A larger number of patients would be required in order to achieve a sample size that is adequately powered for the assessment of AF recurrence. Further randomized studies with larger sample sizes are necessary in order to confirm these results and to establish the relationship between the type of anesthesia used and AF recurrence in this population.

## 6. Conclusions

In patients undergoing HPSD RFCA for AF, the use of GA is associated with improved procedural efficiency, while it is simultaneously associated with an early recurrence rate comparable to MCS. Procedural safety and acute success rates were similar regardless of the type of anesthesia used.

## Figures and Tables

**Table 1:** General and procedural characteristics of the patients.

	GROUP 1 (n = 47)	GROUP 2 (n = 84)	*p*-Value
Age (years old)	58.6 ± 10.6	60.2 ± 10.2	0.41
Gender (males, N, %)	32 (68%)	55 (65.4%)	0.76
Type of AF (paroxysmal, N, %)	34 (72.3%)	68 (80.9%)	0.26
Left atrial diameter (mm)	41.5 ± 5.7	40.2 ± 10.2	0.15
Radiation exposure dosis (μGy)	932.5 [625–1716]	2445 [1228–4791]	<0.0001
Radiation exposure time (min)	4.5 [3–7.1]	7.3 [4.2–13.5]	0.0003
Procedure time (min)	100 [90–120]	160 [130–180]	<0.0001
Number of RF applications	71 [54.8–83.8]	103 [88.5–120.5]	<0.0001
Antiarrhythmic drugs, N (%)			
Amiodarone	20 (42.6)	36 (42.9)	0.97
Propafenone	5 (10.6)	18 (21.4)	0.13
Flecainide	20 (42.6)	29 (34.5)	0.36
Hypertension	27 (57.4)	60 (71.4)	0.11
Diabetes mellitus	9 (19.1)	14 (16.7)	0.72
Ischemic heart disease	6 (12.8)	12 (14.3)	0.81
Heart failure	8 (17)	11 (13.1)	0.54
Mitral regurgitation	4 (8.5)	14 (16.7)	0.20
Obesity	11 (23.4)	23 (27.4)	0.62
COPD	3 (6.4)	6 (7.1)	0.87
Chronic kidney disease	0 (0)	2 (2.4)	0.29
6-month AF recurrence rate, N (%)	10 (21.3)	28 (33.3)	0.15

**Table 2:** Univariable logistic regression for the prediction of recurrence.

Variable	OR	95%CI	*p*
Use of GA	0.55	0.24 to 1.24	0.15
Left atrial diameter (mm)	1.18	1.08 to 1.28	0.0003
Number of RF applications	1.01	1.0009 to 1.03	0.03
Persistent AF	4.47	1.87 to 10.69	0.0007
Amiodarone	2.05	0.95 to 4.40	0.07
Propafenone	0.46	0.15 to 1.45	0.18
Flecainide	0.82	0.37 to 1.81	0.63
Hypertension	1.94	0.82 to 4.58	0.13
Mitral regurgitation	3.79	1.36 to 10.56	0.01

**Table 3:** Multivariable logistic regression for the prediction of recurrence.

	OR	95%CI	*p*
All patients			
Use of GA	0.57	0.19 to 1.70	0.31
Left atrial diameter (mm)	1.09	0.99 to 1.21	0.09
Number of RF applications	1.001	0.98 to 1.02	0.88
Persistent AF	3.33	1.19 to 9.32	0.02
Mitral regurgitation	2.42	0.76 to 7.66	0.13
Paroxysmal AF			
Use of GA	0.34	0.08 to 1.50	0.16
Left atrial diameter (mm)	1.17	1.03 to 1.33	0.01
Number of RF applications	0.99	0.97 to 1.01	0.49
Mitral regurgitation	3.49	0.94 to 12.96	0.06
Persistent AF			
Use of GA	0.80	0.13 to 4.82	0.81
Left atrial diameter (mm)	0.91	0.72 to 1.15	0.44
Number of RF applications	1.02	0.99 to 1.05	0.21
Mitral regurgitation	1.67	0.17 to 16.14	0.66

AF, atrial fibrillation; GA, general anesthesia; LA, left atrial; and RF, radiofrequency.

## Data Availability

Data supporting the findings of this study are available from the corresponding author upon reasonable request.
